# Assessing the impact of GINA guideline–Concordant medication prescribing on asthma outcomes in a tertiary hospital in Saudi Arabia: a retrospective cohort study

**DOI:** 10.3389/fmed.2026.1800158

**Published:** 2026-05-07

**Authors:** Ahmad Alamer, Nazar R. Alrwaili, Mukhtar Alomar, Dhai Aldalgan, Ahmed A. Alanazi, Gadah K. Alonazi, Mohammed M. Alsultan, Saad A. Aldosari

**Affiliations:** 1 Department of Clinical Pharmacy, College of Pharmacy, Prince Sattam Bin Abdulaziz University, Alkharj, Saudi Arabia; 2 Department of Pharmacy, Dr. Sulaiman Al Habib Hospital, Alkhobar, Saudi Arabia; 3 Department of Pharmaceutical Affairs, Dammam Medical Complex, Eastern Health Cluster, Dammam, Saudi Arabia; 4 Department of Pharmacy Practice, College of Pharmacy, Imam Abdulrahman Bin Faisal University, Dammam, Saudi Arabia; 5 Pharmaceutical Care Department, Prince Sattam Bin Abdulaziz University Hospital, Al-Kharj, Saudi Arabia

**Keywords:** asthma, exacerbation, ICS, ICS-formoterol, salbutamol, GINA guideline

## Abstract

**Background:**

Historically, short-acting β2-agonists (SABAs) have been the preferred reliever therapy for asthma. However, their excessive use is associated with poor treatment outcomes, prompting the recent Global Initiative for Asthma (GINA) guidelines to recommend inhaled corticosteroid (ICS)–formoterol as the preferred reliever.

**Objectives:**

This study aimed to assess prescribing patterns of ICS–formoterol and SABAs in asthma patients and to evaluate their impact on clinical outcomes, including exacerbation rates.

**Methods:**

This retrospective cohort study included adult asthma patients (≥18 years) treated at a tertiary hospital in the Eastern Province of Saudi Arabia between July 2022 and July 2023. The primary endpoint was the proportion of patients prescribed either Track 1 (ICS–formoterol) or Track 2 (SABA-based) regimens. The secondary endpoints included asthma exacerbations, oral corticosteroid (OCS) use, and treatment step-ups.

**Results:**

Of the 405 patients included, the mean age (±SD) was 47.8 years (± 16.8) for Track 1 and 51.5 (±15.3) for Track 2. Only 24.4% of the patients were prescribed Track 1 regimens, while 75.6% followed Track 2. Statistically significant differences were observed in the secondary endpoint for asthma exacerbation with an adjusted odds ratio (aOR) of 3.8 (95% CI: 1.12–13.2, *p* = 0.032), favoring Track 1. No statistically significant difference was observed in the need for oral corticosteroids or stepping up in the treatment between the two arms.

**Conclusion:**

Despite guideline recommendations, SABA-based therapy remains predominant in Saudi Arabia and is associated with higher exacerbation rates compared with ICS–formoterol. Knowledge gaps and the complexity of guidelines may contribute to non-adherence. Targeted interventions are needed to align clinical practice with evidence-based recommendations and improve asthma outcomes.

## 1 Introduction

Asthma, a chronic non-communicable disease, is characterized by chronic airway inflammation, variable airflow limitation, and bronchial hyper-responsiveness. It manifests through symptoms such as cough, wheezing, shortness of breath, and chest tightness, which vary in intensity and frequency (1). In 2019, the Global Burden of Disease study estimated that asthma affected 262 million people and caused 455,000 deaths worldwide (2). The pathogenesis of asthma is complex, involving multiple cell types, including T-lymphocytes, B-lymphocytes, mast cells, eosinophils, dendritic cells, and macrophages, which produce inflammatory mediators such as chemokines, cytokines, lipids, and histamine (3). Asthma features early and late inflammatory responses. In the early phase, allergens (e.g., dust mites and pollen) trigger plasma cells to produce immunoglobulin E (IgE), which binds to mast cells and basophils, leading to the release of mediators such as histamines, prostaglandins, leukotrienes, and enzymes (e.g., tryptase). These mediators cause bronchoconstriction, inflammation, and symptoms such as wheezing. Cytokines from mast cells attract additional inflammatory cells, which further increase airway swelling (4). T-helper type 2 cells (Th2), a subset of T-helper cells, release interleukin-4 (IL-4), interleukin-5 (IL-5), interleukin-13 (IL-13), and granulocyte-macrophage colony-stimulating factor (GM-CSF), sustaining inflammation, eosinophil survival, and airway remodeling. IL-13 specifically increases mucus production, nitric oxide levels, and smooth muscle contractility. In the late phase (8–24 h post-exposure), antigen-presenting cells activate T-helper cells, which secrete cytokines to intensify inflammation, while eosinophils produce additional mediators that perpetuate the inflammatory response (5, 6).

Asthma management has evolved with evidence-based guidelines from the Global Initiative for Asthma (GINA) and the Saudi Initiative for Asthma (SINA), which aim to improve diagnosis, treatment, and control (7, 8). Historically, asthma was viewed primarily as a disease of bronchoconstriction, leading to the widespread use of short-acting β2-agonists (SABAs) as the primary reliever therapy. SABAs provide rapid bronchodilation but do not address the underlying inflammation (6). The development of inhaled corticosteroids (ICSs) after the introduction of SABAs shifted treatment toward controlling inflammation (9). Overuse of SABAs, defined as ≥3 canisters (typically 200 puffs each) per year, is associated with increased risks of exacerbations, mortality, and healthcare costs across all asthma severities (7, 10–14). This has prompted a reevaluation of SABAs as a standalone reliever, as they do not reduce airway inflammation, a key driver of asthma morbidity. Large clinical trials, such as SYmbicort Given as needed in Mild Asthma 2 (SYGMA 1) trial, demonstrate that as-needed low-dose ICS–formoterol reduces severe exacerbations by ≥60% in mild asthma compared with SABAs alone, while providing comparable symptom control, lung function, and inflammatory outcomes to daily ICS with as-needed SABAs (14).

The GINA and the SINA have revised their guidelines to prioritize anti-inflammatory reliever therapies over SABA monotherapy (7, 8). In recent updates, the GINA recommends low-dose ICS–formoterol as the preferred reliever for adults and adolescents with mild asthma (treatment Steps 1–2) and as part of maintenance and reliever therapy (MART) for moderate-to-severe asthma (Steps 3–5). These recommendations aim to reduce risks associated with SABA overuse and enhance asthma control through anti-inflammatory treatment. These changes are particularly relevant in regions such as Saudi Arabia, where asthma management faces unique regional challenges.

Despite advances in asthma management, high reliance on SABAs, driven by patient preference for quick-relief inhalers and suboptimal physician adherence to the GINA and SINA guidelines, may lead to poor outcomes, including frequent exacerbations and hospitalizations. To address this gap, this study is, to the best of our knowledge, the first in Saudi Arabia to assess the prescribing patterns of ICS–formoterol and SABAs in asthma patients and to evaluate their impact on clinical outcomes, including exacerbation rates and hospital admissions.

## 2 Methods

### 2.1 Patients/materials and methods

This single-center, retrospective observational cohort study was conducted via chart review at Dammam Medical Complex (DMC), a 423-bed tertiary care hospital in the Eastern Province of Saudi Arabia. DMC provides specialized clinics for the management of asthma patients. The Institutional Review Board (IRB) at DMC approved the study (approval number: PH-22), with a waiver of informed consent due to the retrospective design. The study adhered to the Strengthening the Reporting of Observational Studies in Epidemiology (STROBE) guidelines for reporting (15).

### 2.2 Participants

Eligible patients were adults (aged ≥18 years) diagnosed with asthma [International Classification of Diseases, 10th Revision (ICD-10) codes J45.x] (16) who had an outpatient visit for asthma management at DMC between 1 July 2022 and 31 July 2023. This study period was selected to align with complete electronic medical record (EMR) data availability following the GINA 2019 guidelines. Patients were excluded if they had incomplete medical records, defined as lacking essential data elements critical to the study, including details on prescribed asthma treatments such as reliever or controller therapies, medication dosages, or patient demographic data. Patients were also excluded if they had coexisting chronic respiratory diseases, such as chronic obstructive pulmonary disease, emphysema, bronchitis, cystic fibrosis, or bronchiectasis. We applied a simple random sampling method for selecting patients using Excel’s RAND() function to ensure a representative cohort while managing resource constraints.

### 2.3 Data collection

Baseline characteristics and clinical data were manually extracted from EMRs by trained staff, with a second reviewer verifying more than 50% of the records for accuracy. Data were entered into REDCap in a de-identified manner (17). The extracted data included demographics, such as age, sex, weight, height, and body mass index (BMI); clinical factors, such as asthma severity, smoking status, and comorbid conditions; treatment variables, including reliever and controller therapies; and outcomes, such as exacerbations, corticosteroid use, and step-up therapy.

### 2.4 Definitions

Asthma treatment tracks were defined based on the latest GINA guidelines (7). Track 1 (preferred) was defined as as-needed low-dose ICS–formoterol used as the reliever across Steps 1–5, with maintenance ICS–formoterol as the controller from Step 3 onward, escalating from low dose in Step 3 to high dose with add-on therapies such as long-acting muscarinic antagonists (LAMA) or biologics in Step 5. Track 2 (alternative) was defined as an as-needed SABA used as the reliever across Steps 1–5, with controller therapies ranging from taking ICS whenever SABA was taken in Step 1, to low-dose ICS in Step 2, and to medium−/high-dose ICS combined with a long-acting beta-agonist (LABA), and add-on therapies such as long-acting muscarinic antagonists (LAMA) or biologics in Step 5.

According to the GINA, asthma severity was classified as mild (Steps 1–2, controlled with as-needed ICS–formoterol or low-dose ICS), moderate (Steps 3–4, requiring ICS–LABA), and severe (remains uncontrolled despite optimized treatment with high-dose ICS–LABA or requires high-dose ICS–LABA to prevent it from becoming uncontrolled). Difficult-to-treat asthma was defined as asthma that remains uncontrolled despite medium- or high-dose ICS–LABA treatment or that requires high-dose ICS–LABA to maintain good symptom control and reduce exacerbations.

Adherence to GINA guidelines was defined as prescribing medications in accordance with the patient’s documented asthma severity and assigned GINA treatment track/step. Non-adherence was defined as prescribing patterns that deviated from GINA recommendations, including the use of SABA without concomitant ICS-containing therapy when indicated. Smoking status followed the Centers for Disease Control and Prevention (CDC) criteria: current smoker (≥100 cigarettes, currently smokes), former smoker (≥100 cigarettes, quit), never smoker (<100 cigarettes), or secondhand smoke exposure (exposed to tobacco smoke) (18).

### 2.5 Study outcomes

The primary outcome was provider adherence to the GINA 2024 reliever therapy guidelines, measured as the proportion of patients prescribed as-needed ICS–formoterol (Track 1) or as-needed SABA (Track 2) appropriate to their severity and track. The secondary outcomes included the proportion of patients who had ≥1 asthma exacerbation (ED visit or hospitalization) among those whose providers adhered to or deviated from GINA guidelines, the proportion of patients who were prescribed oral corticosteroids for exacerbations, and the proportion of patients who required step-up therapy (e.g., Step 2 to Step 3) due to inadequate control.

### 2.6 Sample size calculation

We used OpenEpi (https://www.OpenEpi.com) for sample size calculation: a minimum sample size of 351 was required for the study, assuming an estimated proportion of 50% of patients on Track 1, with a 5% margin of precision and an alpha level of 0.05.

### 2.7 Statistical analysis

**Figure 1:**
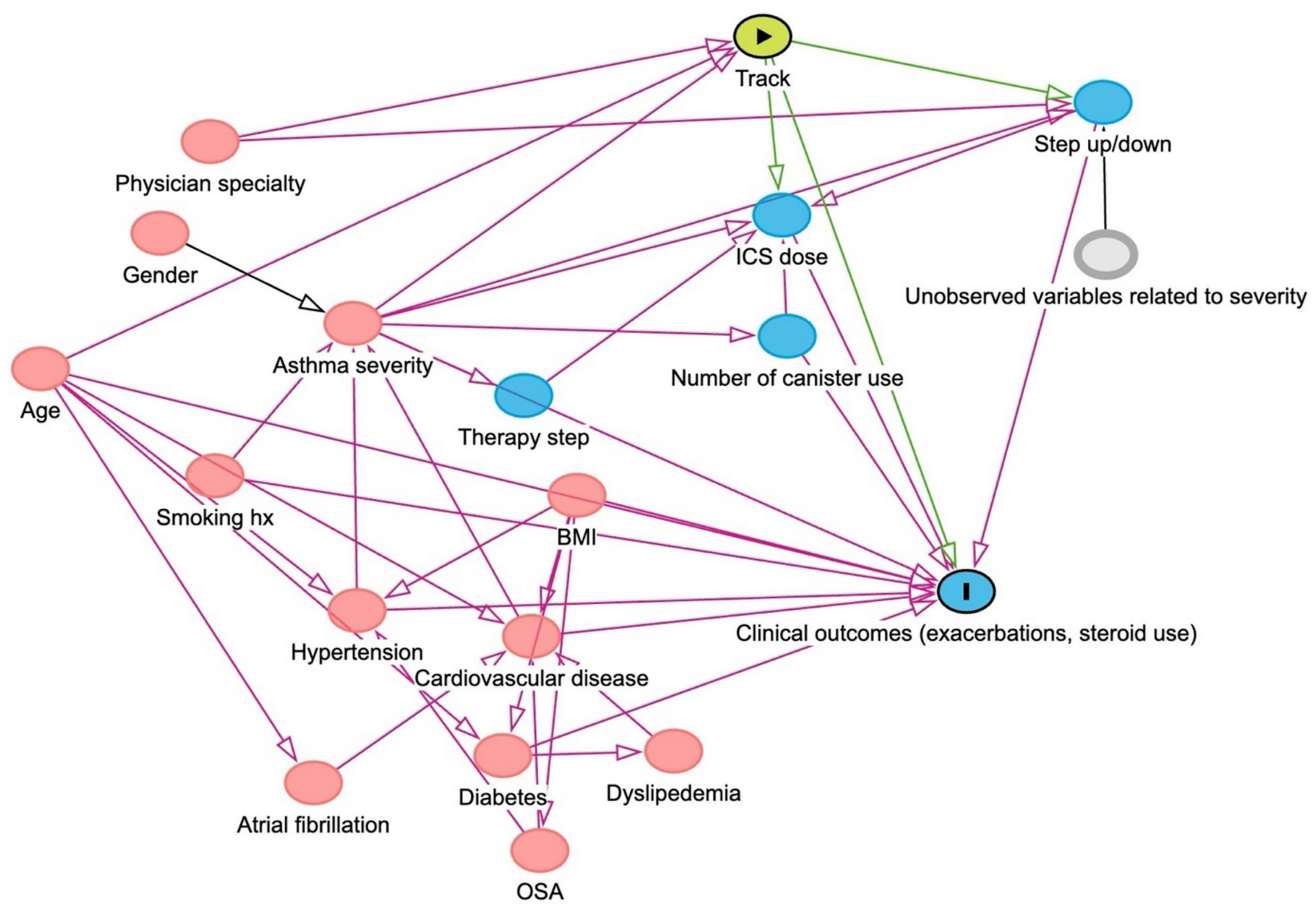
Directed acyclic graph (DAG) drawing the relationship between variables, exposure of interest, and clinical outcomes.

Descriptive statistics summarized patient characteristics, including age, sex, asthma severity, smoking status, treatment track (Track 1 or Track 2), body mass index (BMI), and comorbidities (allergic rhinitis, atopic dermatitis, obstructive sleep apnea, psychiatric disorders, gastroesophageal reflux disease [GERD], hypertension, ischemic heart disease, atrial fibrillation, diabetes mellitus, nasal polyps, pulmonary hypertension, and dyslipidemia). Continuous variables were reported as means and standard deviations, and categorical variables were reported as frequencies and percentages. The primary outcome, provider adherence to GINA report reliever therapy recommendations, was expressed as a frequency and a percentage. The secondary outcomes, including the proportion of patients who had ≥1 asthma exacerbation (an emergency department visit or hospitalization due to asthma), who were prescribed oral corticosteroids, and who required step-up therapy (escalation in GINA treatment step, e.g., from Step 2 to Step 3), were compared between the Track 1 and Track 2 groups using multivariable logistic regression models with a binomial distribution and logit link that included a minimum adjustment set derived from a directed acyclic graph (DAG) (Figure 1). There was no substantial missing data; therefore, a complete-case analysis was applied. Analyses were conducted using R (version 4.3.2).

## 3 Results

### 3.1 Study population and characteristics

**Figure 2:**
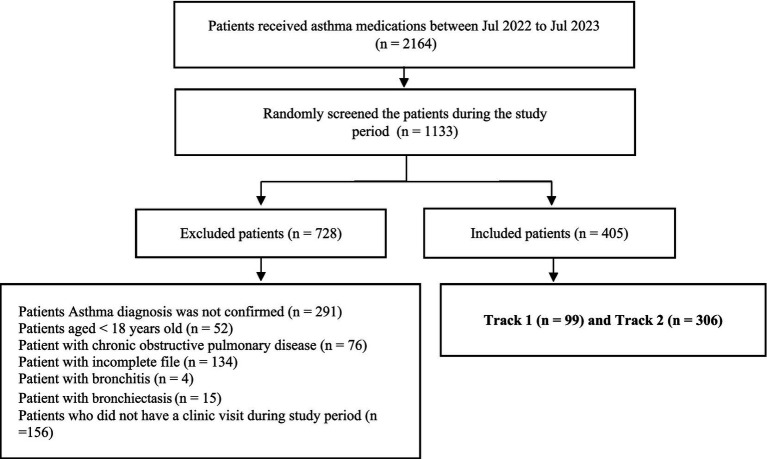
Patient selection flowchart.

From July 2022 to July 2023, 2,164 patients receiving asthma medications were eligible for screening in this retrospective cohort study. Of the 1,133 patients randomly selected, 728 were excluded due to incomplete medical records or coexisting chronic respiratory diseases, leaving 405 patients: 99 (24.4%) were prescribed Track 1 treatment, and 306 (75.6%) were prescribed Track 2 treatment, as shown in Figure 2.

Demographic and clinical characteristics are summarized in Table 1. Track 2 patients were older (51.5 ± 15.3 vs. 47.8 ± 16.8 years, *p* = 0.030). The mean BMI was slightly higher in Track 2 (32.9 kg/m^2^ vs. 31.4 kg/m^2^, *p* = 0.115), while the smoking rate was lower in Track 2 patients, although this difference was not statistically significant (11.8% vs. 14.3%, *p* = 0.540).

**Table 1:** Baseline characteristics of asthma patients by GINA treatment track (*N* = 405).

Characteristics	Track 1 (ICS–formoterol) (*n* = 99)	Track 2 (SABA-based) (*n* = 306)	*p*-value
Demographics			
Age, years, mean ± SD	47.8 ± 16.8	51.5 ± 15.3	0.030
BMI, kg/m^2^, mean ± SD	31.4 ± 8.6	32.9 ± 8.8	0.115
Female, *n* (%)	68 (68.7)	223 (72.9)	0.498
Non-Saudi, *n* (%)	3 (3.0)	18 (5.9)	0.394
Comorbidities, *n* (%)			
Allergic rhinitis	44 (44.4)	151 (49.3)	0.464
Atopic dermatitis	1 (1.0)	7 (2.3)	0.705
Obstructive sleep apnea	5 (5.1)	14 (4.6)	1.000
Psychiatric disorders	0 (0.0)	3 (1.0)	0.753
GERD	16 (16.2)	53 (17.3)	0.910
Hypertension	26 (26.3)	86 (28.1)	0.820
Ischemic heart disease	13 (13.1)	20 (6.5)	0.061
Atrial fibrillation	2 (2.0)	10 (3.3)	0.768
Diabetes mellitus	22 (22.2)	71 (23.2)	0.949
Nasal polyps	2 (2.0)	1 (0.3)	0.301
Pulmonary hypertension	0 (0.0)	1 (0.3)	1.000
Dyslipidemia	8 (8.1)	16 (5.2)	0.424
Smoking status, *n* (%)			0.540
Current/former smoker	14 (14.3)	36 (11.8)	
Non-smoker (never/passive/unknown)	85 (85.9)	270 (88.2)	
Specialty care, *n* (%)			
Pulmonology (asthma clinic)	97 (98.0)	297 (97.1)	
Internal medicine	1 (1.0)	1 (0.3)	
Home healthcare	1 (1.0)	8 (2.6)	
Controller medications, *n* (%)			
Budesonide–formoterol DPI, 160 mcg/4.5 mcg	97 (98.0)	169 (55.2)	<0.001
Fluticasone–salmeterol, 250 mcg	1 (1.0)	52 (17.0)	<0.001
Fluticasone furoate–vilanterol, 200 mcg/25 mcg	1 (1.0)	83 (27.1)	<0.001
Fluticasone, 125 mcg MDI	0 (0.0)	2 (0.7)	1.000
ICS daily dose, *n* (%)			<0.001
Low dose	17 (17.2)	5 (1.6)	
Medium dose	80 (80.8)	203 (66.3)	
High dose	2 (2.0)	98 (32.0)	
Other controller medications, *n* (%)			
Montelukast (LTRA)	0 (0.0)	10 (3.3)	0.147
Tiotropium (LAMA)	4 (4.0)	72 (23.5)	<0.001
Azithromycin	0 (0.0)	1 (0.3)	1.000
Dupilumab (anti-IL-4R)	0 (0.0)	1 (0.3)	1.000
GINA step classification, *n* (%)			
Step 1	1 (1.0)	1 (0.3)	
Step 2	0 (0.0)	1 (0.3)	
Step 3	17 (17.2)	2 (0.7)	
Step 4	80 (80.8)	294 (96.1)	
Step 5	1 (1.0)	8 (2.6)	
Asthma severity, *n* (%)			0.045
Mild	1 (1.0)	2 (0.7)	
Moderate	95 (96.0)	264 (86.3)	
Severe	1 (1.0)	17 (5.6)	
Difficult to treat	2 (2.0)	23 (7.5)	

Data are presented as mean ± standard deviation (SD) or n (%). *p*-values were calculated using *t*-tests for continuous variables, chi-square tests for categorical variables, or Fisher’s exact test for small cell sizes (e.g., atopic dermatitis). *p*-values were not calculated for specialty care and GINA step classification due to small sample sizes. Track 1 represents patients prescribed ICS–formoterol as a reliever and maintenance; Track 2 represents SABA as a reliever with ICS or ICS–LABA maintenance. Psychiatric disorders include schizophrenia, drug addiction, depression, and unspecified disorders. BMI, body mass index; GERD, gastroesophageal reflux disease; ICS, inhaled corticosteroid; LTRA, leukotriene receptor antagonist; LAMA, long-acting muscarinic antagonist; MDI, metered-dose inhaler; DPI, dry powder inhaler. *p < 0.05 indicates statistical significance.

Comorbidities, including allergic rhinitis (44.4% vs. 49.3%), hypertension (26.3% vs. 28.1%), and diabetes (22.2% vs. 23.2%), showed no significant differences (*p* > 0.05), although ischemic heart disease trended higher in Track 1 (13.1% vs. 6.5%, *p* = 0.061). The majority of patients attended pulmonology clinics (98.0% Track 1, 97.1% Track 2, *p*-values not reported for cell counts <5). Controller medication use varied: Track 1 patients predominantly used budesonide–formoterol (98.0%), while Track 2 patients used diverse ICS–LABA combinations, with budesonide–formoterol being the most common at 55.2%, *p* < 0.001. Track 1 used more low- and medium-dose ICS (17.2 and 80.8%, respectively), whereas Track 2 used more medium- and high-dose ICS (66.3 and 32.0%, respectively). Tiotropium use was higher in Track 2 (23.5% vs. 4.0%, *p* < 0.001). GINA Step 4 was more common in Track 2 (96.1% vs. 80.8%), while Step 3 was more common in Track 1 (17.2% vs. 0.7%). Track 2 had more severe (5.6% vs. 1.0%) and difficult-to-treat asthma (7.5% vs. 2.0%), while Track 1 had more moderate cases (96.0% vs. 86.3%, *p* = 0.045).

### 3.2 Clinical outcomes

Table 2 presents asthma-related outcomes for Track 1 and Track 2. Asthma exacerbations occurred in 3.0% (3/99) of Track 1 patients and 12.7% (39/306) of Track 2 patients, with an unadjusted odds ratio (OR) of 4.67 (95% CI: 1.41–15.48, *p* = 0.012). and an adjusted OR of 3.8 (95% CI: 1.12 – 13.2, *p* = 0.032) after adjusting for age, smoking status, asthma severity, diabetes, hypertension, and cardiovascular diseases, indicating a statistically significant higher risk in Track 2. The E-value was calculated for this outcome and was found to be 7.06. Oral corticosteroid prescriptions were reported in 2.0% (2/99) of Track 1 patients and 4.9% (15/306) of Track 2 patients, with an unadjusted OR of 2.5 (95% CI: 0.56–11.12, *p* = 0.229) and an adjusted OR of 2.17 (95% CI: 0.45–10.42, *p* = 0.332) after adjusting for the same covariates. Step-up therapy was required in 1.0% (1/99) of Track 1 patients and 5.9% (18/306) of Track 2 patients, with an unadjusted OR of 6.13 (95% CI 0.80 – 46.48, *p* = 0.080) but a non-significant adjusted OR of 4.8 (95% CI: 0.62 – 37.12, *p* = 0.131) after adjusting for age and asthma severity due to limited sample size. Overall, Track 2 had a significantly higher incidence of asthma exacerbations, but differences in oral corticosteroid use and step-up therapy were not statistically significant after adjustments.

**Table 2:** Asthma-related clinical outcomes: Track 1 vs. Track 2.

Event	Track 1 (*n* = 99)	Track 2 (*n* = 306)	Unadjusted OR (95% CI, *p*-value)	Adjusted OR (95% CI, *p*-value)
Exacerbations	3 (3.0%)	39 (12.7%)	4.67 (1.41–15.48, *p* = 0.012)	3.8 (1.12–13.2, *p* = 0.032)
Need for oral corticosteroids	2 (2.0%)	15 (4.9%)	2.5 (0.56–11.12, *p* = 0.229)	2.17 (0.45–10.42, *p* = 0.332)
Stepped-up therapy	1 (1.0%)	18 (5.9%)	6.13 (0.80–46.48, *p* = 0.080)	4.8 (0.62–37.12, *p* = 0.131)

Reference level is Track 1. Asthma exacerbation and need for oral corticosteroids were adjusted for age, asthma severity, smoking status, diabetes, hypertension, and cardiovascular diseases. Stepped-up therapy was adjusted for age and asthma severity. OR, odds ratio; CI, confidence interval. The E-value was calculated for the exacerbation outcome and was found to be 7.06. This indicates that an unmeasured confounder would need to be associated with both the exposure and the exacerbation outcome by a risk ratio of at least 7.06 to fully explain away the observed association.

## 4 Discussion

To the best of our knowledge, this is the first study in Saudi Arabia to evaluate prescribing patterns and clinical outcomes following the GINA guideline updates in the real-world setting, which recommend low-dose ICS–formoterol as the preferred reliever therapy across all treatment steps for adults and adolescents aged ≥12 years with asthma (7). Among our cohort of asthma patients, only a small percentage were prescribed Track 1 therapy, while the majority of patients received Track 2 therapy. Comparatively, a study from the United States reported even lower adherence to ICS–formoterol. Among the 127 asthma patients discharged post-exacerbation, only 2.4% (3/127) received ICS–formoterol as a reliever, while 76.4% (97/127) were prescribed a short-acting beta-agonist (SABA) inhaler alongside ICS-containing controller therapy. Notably, 82% of patients (92/112) were discharged with ICS–LABA inhalers as controller therapies, with fluticasone–salmeterol being the most common (41%) (19). These patients represent an ideal population for using ICS–formoterol as a reliever.

In addition, our study found that patients in Track 2 experienced significantly more asthma exacerbations, defined as ≥1 emergency department visit or hospitalization, even after adjusting for confounders. However, differences in oral corticosteroid prescriptions and step-up therapy were not significant, although trends suggest a potential clinical benefit for Track 1. Non-significant differences in corticosteroid use and step-up therapy may be attributed to the small Track 1 sample size (n = 99) or low event rates, limiting statistical power. These findings align with evidence from the SYGMA 1 and SYGMA 2 trials, which demonstrated the superiority of as-needed budesonide–formoterol over SABA-based regimens (14, 20). In SYGMA 1 (*N* = 3,836), patients using as-needed budesonide–formoterol had more weeks of well-controlled asthma (34.4% vs. 31.1% in the terbutaline group, 95% CI: 1–1.3, *p* = 0.046) and lower annual exacerbation rates (0.07 vs. 0.20, *p* < 0.001) (14). Similarly, SYGMA 2 (*N* = 4,176) showed that as-needed budesonide–formoterol was non-inferior to maintenance budesonide with as-needed SABA in reducing severe exacerbations (rate ratio 0.97, upper one-sided 95% confidence limit, 1.16). It is worth noting that, unlike our study population, which primarily consisted of patients with moderate-to-severe asthma (Steps 3–4), the SYGMA trials included patients with mild asthma (Steps 1–2).

Our observational study findings align with broader research on asthma management and guideline adherence. For instance, a cross-sectional survey of 300 healthcare professionals in Jordan, conducted between May and June 2023, found that 47% were aware of the GINA guideline updates. For patients with mild asthma, 20.3% of healthcare professionals would prescribe as-needed low-dose ICS therapy, such as budesonide and formoterol, as needed. For patients with moderate asthma, only 15.7% would prescribe as-needed low-dose ICS, such as budesonide and formoterol (21). Similarly, a study by Chapman et al. found that, among 736 physicians in Australia, Canada, China, and the Philippines, 13% (96/736) would prescribe ICS–formoterol maintenance and reliever therapy (MART) for patients with mild asthma, while 47.4% (349/736) would opt for inhaled SABAs alone, such as salbutamol. For patients with moderate asthma, 32.8% (241/735) of physicians would prescribe an ICS–LABA fixed-dose combination alongside an inhaled SABA, 28.8% (212/735) would choose low-dose ICS with an inhaled SABA, and 16.2% (119/735) would consider ICS–formoterol MART (22). These findings indicate low adoption of guideline-recommended preferred reliever therapy.

Given the significant non-adherence to GINA-recommended Track 1 reliever therapy, exploring barriers to guideline implementation is necessary but could not be conducted in this study due to its retrospective nature of the study. Studies highlight that patient and provider knowledge significantly affect guideline adherence (23–25). For instance, a cross-sectional study of 27 emergency physicians found that physicians with adequate guideline knowledge were more likely to comply with the 2011 GINA treatment guidelines (23). Moreover, Holst et al. identified patients’ lack of knowledge as a key barrier to adopting GINA-recommended therapy (25). The presence of multiple asthma guidelines and the frequency of their updates can also create confusion among healthcare providers and hinder consistent implementation. In addition to GINA, other guidelines, such as those from the National Asthma Education and Prevention Program (NAEPP) in the United States, may present overlapping or conflicting recommendations, resulting in variability in prescribing practices. For instance, while the GINA emphasizes as-needed ICS–formoterol for all asthma severities, NAEPP guidelines still endorse as-needed SABA-based regimens (26). This divergence can complicate decision-making for providers, particularly in regions with diverse healthcare systems or limited access to updated training. Moreover, the frequency of guideline updates is another obstacle. Potential strategies, such as integrating guideline updates into electronic health record systems or providing regular, concise training modules, could help mitigate these challenges.

In a local context, a systematic review by Alqahtani et al. (27) reviewed 17 studies conducted in Saudi Arabia for asthma control. Only one relevant study was identified that was conducted by AL-Jahdali (28), in which a fixed combination of ICS and LABA was found to be associated with better asthma control, as assessed by physicians according to GINA guidelines (OR = 1.77; 95% CI: 1.29–2.44). In addition, a study by Al-Jahdali (29) revealed that inhaler misuse was observed in 45% of patients and was significantly associated with worse asthma control, as defined by the Asthma Control Test ≤ 15. The overprescription of SABA reliever use was further studied in a Saudi Arabian cohort as part of SABINA III [part of the global SABINA (SABA use in Asthma) real-world study] (30). The study included seven sites in Saudi Arabia guided by an older version of the GINA guidelines (2017). There was a low prescription rate for ICS among the studied population, with only 5.0, while 60.6% of patients in Saudi Arabia were overprescribed SABA (defined as ≥3 SABA canisters in the 12 months prior to the study) (30). The impact of asthma care tracks (Track 1 vs. Track 2) was not studied by this study, as this was not part of the standard of care at that time.

Our study has several strengths. First, it provides real-world evidence comparing GINA Track 1 and Track 2 regimens in terms of clinical outcomes. Second, it includes a moderate sample size derived through random sampling to minimize bias. However, the study has several limitations. First, the retrospective design may introduce selection bias, missing data, documentation bias, lack of randomization, and potential unmeasured confounders, thereby limiting causal inference. Second, the study was conducted at a single tertiary hospital, which may limit the generalizability of results to other regions or care settings in Saudi Arabia, as the reliance on electronic health records may introduce documentation bias. Third, there was potential underreporting of over-the-counter SABA use. Additionally, classification into Tracks 1 and 2 was based on prescribing patterns rather than confirmed patient adherence, which may influence outcomes. Although DAGs were used to identify confounding bias, the method is not without limitations. The E-value obtained was 7.06, indicating the magnitude of association that an unmeasured confounder would need to have with both the exposure and the outcome to explain away the findings for the exacerbation outcome. We note that lung function tests were not readily available in our data. According to the GINA guidelines, pulmonary function tests are primarily used to confirm the diagnosis, and once the diagnosis is confirmed, these tests are mainly useful for predicting future risk and assessing airflow limitation. In addition, validated tools, including the Asthma Control Test (ACT) and the Asthma Control Questionnaire (ACQ), for assessing asthma control during patient visits were not available to us. However, neither the ACT nor the ACQ has been validated in patients receiving ICS–formoterol or ICS–SABA as reliever therapy. Furthermore, these tools do not assess asthma control over an extended period, such as 12 months; rather, they are better suited to assessing short-term asthma control over a few weeks (7). Therefore, we believe that asthma exacerbation—defined as an emergency department visit or hospitalization due to asthma oral corticosteroid prescriptions, and step-up therapy (i.e., escalation in GINA treatment step) are better indicators for asthma control over a long period of time. Finally, the higher exacerbation rate in Track 2 compared to Track 1 cannot be fully explained in our model due to the potential for residual unmeasured confounding, such as the absence of fractional exhaled nitric oxide (FeNO) or inflammatory phenotype biomarkers, such as blood eosinophil counts, and overlap syndromes with COPD.

## 5 Conclusion

This study highlights a significant discrepancy between clinical practice and GINA recommendations in Saudi Arabia, with persistent reliance on SABA despite evidence supporting the superiority of ICS–formoterol; however, these findings should be interpreted with caution due to the study design.

The higher exacerbation rates in Track 2, combined with barriers such as knowledge gaps and the complexity of multiple guidelines, underscore the urgent need for strategies to align practice with evidence-based recommendations. Future research should investigate these barriers and evaluate interventions such as enhanced provider training, patient education, and guideline harmonization—to promote ICS–formoterol adoption across diverse healthcare settings in Saudi Arabia, the US, and globally.

## Data Availability

The original contributions presented in the study are included in the article/supplementary material, further inquiries can be directed to the corresponding author.
